# Children’s GPS-determined versus self-reported transport in leisure time and associations with parental perceptions of the neighborhood environment

**DOI:** 10.1186/s12942-016-0045-9

**Published:** 2016-05-05

**Authors:** Griet Vanwolleghem, Jasper Schipperijn, Freja Gheysen, Greet Cardon, Ilse De Bourdeaudhuij, Delfien Van Dyck

**Affiliations:** Department of Movement and Sport Sciences, Faculty of Medicine and Health Sciences, Ghent University, Watersportlaan 2, 9000 Ghent, Belgium; Research Unit for Active Living, Department of Sport Science and Clinical Biomechanics, University of Southern Denmark, Odense, Denmark; Research Foundation Flanders (FWO), Egmontstraat 5, 1000 Brussels, Belgium

**Keywords:** GPS, Transport in leisure time, Children, Physical environment

## Abstract

**Background:**

This study aimed to examine both GPS-determined and self-reported walking, cycling and passive transport in leisure time during week- and weekend-days among 10 to 12-year old children. Comparisons between GPS-determined and self-reported transport in leisure time were investigated. Second, associations between parental perceptions of the neighborhood environment and GPS-determined walking, cycling and passive transport in leisure time were studied.

**Methods:**

Children (10 to 12-years old; n = 126) wore a GPS device and an accelerometer for 7 consecutive days to assess objectively measured transport in leisure time and filled out a diary to assess self-reported transport in leisure time. Parents completed a questionnaire to assess parental perceptions of the neighborhood environment. Pearson correlations and t-tests were used to test for concurrent validity and differences between GPS-determined and self-reported transport in leisure time. Generalized linear models were used to determine the associations between the parental perceptions of the neighborhood environment and GPS-determined transport in leisure time.

**Results:**

Overall, children under-reported their walking and cycling in leisure time, compared to GPS-determined measures (all p values <0.001). However, children reported their passive transport in leisure time during weekend days quite accurate. GPS-determined measures revealed that children walked most during weekdays (M = 3.96 trips/day; 26.10 min/day) and used passive transport more frequently during weekend days (M = 2.12 trips/day; 31.39 min/day). Only a few parental perceived environmental attributes of the neighborhood (i.e. residential density, land use mix access, quality and availability of walking and cycling facilities, and aesthetics) were significantly associated with children’s GPS-determined walking, cycling or passive transport in leisure time.

**Conclusions:**

To accurately assess children’s active transport in leisure time, GPS measures are recommended over self-reports. More research using GPS with a focus on children’s transport in leisure time and investigating the associations with parental perceptions of the neighborhood environment is needed to confirm the results of the present study.

**Electronic supplementary material:**

The online version of this article (doi:10.1186/s12942-016-0045-9) contains supplementary material, which is available to authorized users.

## Background

Physical activity provides numerous health benefits for children’s physical and mental functioning [1, 2]. Engagement in active transport (walking and cycling) can offer an important contribution to daily physical activity levels of 6 to 12-year olds [3–5]. Transport to school and in leisure time (i.e. to other destinations besides school) are indicated as important travel purposes [6, 7]. Despite the well-known benefits of active transport, many primary schoolchildren do not walk or cycle to leisure-time destinations [8–11]. Among 10 to 13-year old Flemish children (northern part of Belgium), who often live within active transport feasible distances from leisure-time destinations [12], 41 % of children’s trips per day during leisure time are passive (dropped off by car, using public transport). Since independent mobility increases from the age of ten and children’s choice of active transport mode becomes more important to travel independently [3, 13], children in their last years of primary school (10 to 12-year old) are an important target group to promote active transport in leisure time.

To develop effective interventions, insight into the determinants of children’s context-specific active transport (e.g. active transport in leisure time) is needed to target the specific active transport behavior [14, 15]. However, only a few studies examined children’s active transport during leisure time [5, 8, 9, 16–19]. Factors influencing children’s active transport in leisure time may be different than those influencing active transport to school [5], since transport in leisure time is less mandatory and involves less time constraints [8]. Since children’s time in out-of-home activities in leisure time is known to differ between week- and weekend-days [7, 18], it is important to gain a clear understanding of children’s transport in leisure time during both week- and weekend-days. To gain insight into the determinants of active transport in leisure time, the socio-ecological model developed by Sallis et al. identified correlates at multiple levels (individual, social and physical environment), related to specific domains of physical activity [20]. Specifically, there is growing interest in examining the relationship between the physical environment and active transport in leisure time in primary schoolchildren [16–18]. For children, the neighborhood environment is important, given that children’s active transport mostly takes place in a neighborhood context [15]. Furthermore, previous studies identified perceived frequency and quality of walking and cycling facilities [8, 9], good road connectivity [9], access to destinations [9] and presence of green space [16, 17] in the neighborhood as important determinants of children’s active transport in leisure time. Since walking and cycling are two different activities with different determinants, research should make a distinction between both activities [14, 16]. However, only a few studies reported specific results for walking and cycling separately [16, 18, 19]. Next to the objective neighborhood environment, the parental perceptions of the neighborhood environment are of importance because parents still play a role to let their child walk or cycle independently despite children’s increase of independent mobility [21]. Additionally, clear knowledge of the environmental perceptions of the neighborhood that motivate parents to select a passive transport mode can be relevant when developing interventions promoting children’s active transport. Until now, only a limited number of studies included measures of passive transport [8, 18, 22]. Therefore, the focus of the present study is on 10 to 12-year old children’s active (walking and cycling) and passive transport in leisure time during week- and weekend-days and the association with parental perceptions of the neighborhood environment.

Up to now, children’s active transport in leisure time has mainly been assessed by self-reported questionnaires [11, 16, 23], frequently resulting in bias and conflicting findings [24–26]. In particular, reporting transport in leisure time adequately is difficult, especially for children, since a specific context is required and they may not always accurately remember their transport mode and number of actual trips. But also parent-reported transport holds limitations as parents do not always accurately remember their child’s transport in leisure time, especially not for short and occasional transport (e.g. combined trip with public transport and walking) [26]. Consequently, an objective method to assess children’s transport in leisure time (i.e. transport mode, number and duration of trips) is preferable to study the determinants of the specific transport behavior more accurately. Recently, Global Positioning Systems (GPS) have been increasingly used to assess transport behavior in a specific outdoor context. They provide accurate measures of transport distances [27–29] and speed [29–31] and a distinction between walking, cycling and passive transport can be made. Combined with geographical information (e.g. school address to exclude transport to school), GPS-data can be used to objectively assess the mode of transport in a context-specific physical activity. Additionally, it is also possible to accurately assess the duration of the context-specific physical activity [29, 32, 33]. So this innovative method may offer a suitable solution to objectively and accurately assess children’s (time spent in) active and passive transport providing a clear advantage compared to the previously used self-reported questionnaires. To date, limited information is available with respect to the objective measures of children’s transport in leisure time [34]. Larouche et al. [34] emphasized in a recent review that further research on the concurrent validity between children’s GPS-determined and self-reported transport in leisure time is needed. Furthermore, it is unclear if children over- or underreport transport in leisure time compared to GPS-determined measures. Additionally, only one study reported results of children’s GPS-determined transport in leisure time on both week- and weekend-days [35].

To summarize, there is a lack of knowledge of children’s objectively GPS-determined transport (i.e. active/passive transport mode, number and duration of trips) in leisure time compared to self-reported transport [34, 36, 37]. Current literature [34–39] also lacks knowledge about how GPS-determined transport in leisure time during week- and weekend-days is associated with parental perceptions of the neighborhood environment. Therefore, the first aim of the present study was to compare GPS-determined with self-reported walking, cycling and passive transport in leisure time among children. We hypothesized that children would under-report their transport in leisure time compared to GPS-determined measures. The second aim of the present study was to examine the associations between the parental perceptions of the neighborhood environment and GPS-determined walking, cycling and passive transport in leisure time. Since we hypothesized that GPS-determined and/or self-reported transport in leisure time would differ between week- and weekend-days, analyses were stratified on week- and weekend-days.

## Methods

### Participants and procedure

In October 2013, a convenience sample of eight primary schools in Flanders (northern part of Belgium) in two regions (East- and West-Flanders) was contacted by phone and four primary schools agreed to participate (two located in a suburban area, 150–500 residents/km^2^ (total number of pupils = 235), two located in an urban area, >500 residents/km^2^ (total number of pupils = 295).

Primary schoolchildren attending 5th and 6th grade (10–12 year old) (n = 270) were invited to participate in the study. The study was conducted in the winter of 2013–2014 (December 2013–January 2014) in Flanders. Conducting the study in the winter had no significant influence on children’s transport measures since Flanders has mild winters. Parental informed consent for children to wear an accelerometer and GPS device was obtained from the parents of 188 children (70 %). The measurement period lasted 1 week, including two weekend days. Children wore an accelerometer and GPS device to assess objectively measured transport in leisure time. Additionally, they filled out a diary, together with their parents, to assess self-reported transport in leisure time. Complete diaries were received from 144 children (77 %). Additionally, parents of the children (n = 188) were asked to complete a questionnaire including socio-demographic information and parental perceptions of the neighborhood environment. In total, 172 parents (91 %) completed the parental questionnaire [suburban schools (n = 94), urban schools (n = 78)]. Measurement instruments, diaries and parental questionnaires were distributed and collected at the schools. A researcher went to the different classes with participating children and explained the purpose of the study, demonstrated how to wear both measurement instruments correctly and emphasized practical issues (e.g. importance of recharging GPS device at night, filling out the diary correctly). The present study was approved by the Ghent University Ethics Committee (EC UZG 2013/228).

### Measurements

#### Socio-demographic information

The first section of the parental questionnaire contained general questions about the child (age, sex) and the parents (educational level of parents), to obtain socio-demographic information. Educational level of the parents was used as a proxy measure of children’s socio-economic status (SES). The educational level was based on four options: did not complete secondary school, completed secondary school, completed college, or completed university. Children were identified as being of high SES when at least one parent reached a college or university level.

#### Parental perceptions of the neighborhood environment

A second part of the parental questionnaire contained questions to assess perceived neighborhood environmental attributes. Some questions were taken from the parent version of the Neighborhood Environmental Walkability Scale for Youth (NEWS-Y) [9] and other questions were added, to comply with the Belgian environment (see Additional file 1 for outline questionnaire). Seven subscales were included and calculated: (1) residential density (presence of different types of residences (e.g. separate or standalone one family homes, connected townhouses or row houses, apartments), (2) land use mix access (access to neighborhood services (e.g. ease to walk to public transport, possibilities to do shopping in a local area)), (3) street network connectivity (connectedness of street network (e.g. presence of intersections, dead-end streets and alternate routes), (4) availability and quality of walking and cycling facilities (e.g. presence and maintenance of sidewalks/cycling lanes in most streets), (5) aesthetics (presence of aesthetic features (e.g. green spaces)), (6) perceived safety from traffic (e.g. speed of traffic in neighborhood) and (7) perceived safety from crime (e.g. crime prevalence in the neighborhood). Each subscale contained multiple questions (see Additional file 1 for the questions with corresponding response options). Response options for the three questions to obtain the subscale residential density were scored on a 5-point scale, ranging from none to all. Since connected townhouses, row houses and apartments are considered to be more person-dense than separate or standalone one family homes, the residential density items were weighted relative to the average density of separate or standalone one family homes [40]. The subscale residential density was then calculated by the following formula: score on question 1a (separate or standalone one family homes) + 12*score on question 1b (connected townhouses or row houses) + 25*score on question 1c (apartments) [41]. Response options for the questions regarding the other subscales were scored on a 4-point scale, ranging from strongly disagree to strongly agree. Those subscales were scored by taking the mean of the different question scores. Internal consistency for all subscales of the questionnaire used in this study was found to be acceptable.

#### Self-reported transport in leisure time

To assess self-reported transport in leisure time, children (together with their parents) were asked to report daily on their trips per day in a diary during the measurement period. They were asked to report all trips that lasted at least 3 min and to report also combined transport (e.g. a trip including public transport and walking to a bus stop). For each trip, they were asked to report the transport mode (walking, cycling, car, public transport). Children were also asked to report what trips were to and from school. Those trips could be excluded for further analyses. Trips per day for walking, cycling or passive transport in leisure time were used as main outcomes. The main outcomes were stratified in week- and weekend-days.

#### GPS-determined transport in leisure time

Children were asked to wear a GPS device QStarz BT-Q1000XT (Qstarz International Co., Ltd, Taipei, Taiwan) and an Actigraph accelerometer GT1 M or GT3X (Actigraph MTI, Manufacturing Technology Inc., Pensacola, FL, USA) to objectively assess their transport in leisure time. The QStarz BT-Q1000XT GPS device recorded location and speed. The speed was used to obtain children’s transport mode. The accelerometer data was only used to determine device wear time in the analyses presented in this paper. The QStarz unit has demonstrated a good inter-unit reliability [31, 42, 43] and a median dynamic positional error of 2.9 m [28]. Children wore the devices on a belt on the hip (opposite sides) during seven consecutive days, including two weekend days [44]. Children were asked to wear the accelerometer and GPS device during waking hours and to remove the instruments for aquatic activities (e.g. swimming, showering) and for activities that prohibit the instruments (e.g. contact sports). Children were asked to charge the GPS every night. Accelerometers and GPS devices were set to record data every 15-s. Processed GPS data were matched to accelerometer data in 15-s epochs using PALMS (Personal Activity and Location Measurement Systems) [45, 46].

Children with a minimum of 9 h of combined accelerometer and GPS data on at least 4 days (including at least one weekend day) were included in the analyses (similar to [33, 47]). Data from day 1 were excluded from the analyses because the instruments were handed out at different times during the first day, resulting in less than 9 h of wear time for day 1. Additionally, non-wear time was defined as 60 min or more of zero values [48]. Due to insufficient wear time, invalid wear days and technical problems (e.g. signal loss, no corresponding GPS and accelerometer data), data from 62 children (33 %) were excluded from the analyses. In total, 126 children had valid combined accelerometer and GPS data. The demographic characteristics (age, sex, SES and school location) of the included children (n = 126) were comparable (all p-values of the χ^2^- and t-tests ≥ 0.05) with those of the sample of children who dropped out (n = 62).

### Data processing of GPS-data

PALMS combined the activity data (accelerometers) with the location data (GPS) and it identified and classified children’s GPS-determined transport. Based on the validation trip and trip mode detection algorithms developed by Carlson et al. [49], a trip was defined as a continuous period of movement with the same mode of transportation for at least 3 min, allowing for stationary periods of maximum 5 min [49]. Additionally, PALMS classified children’s trips into walking, cycling and passive (vehicle) transport based on the speed (walking: 1 to <10 km/h; cycling: 10 to <25 km/h; passive transport: ≥25 km/h) [49].

A purpose built PostgreSQL database was used to combine the PALMS dataset (combined accelerometer and GPS data at 15 s epoch) with digital geographical data (e.g. the road network) and information on school schedules, to calculate the specific outcome variables. Transport in leisure time was defined as all transport outside school hours during weekdays and all trips in the weekend, excluding all trips to and from school. Outside school hours during weekdays was defined as the period before school starts and after school ends, which was slightly different for each school. In Belgium, most primary schools start between 8:15 and 8:30 A.M. and run until 15:30–16:00 P.M., except for Wednesdays. On Wednesdays, Belgian primary schools run until 12:00 PM. The specific time schedule of each school was used to identify leisure time during weekdays. Using the school and home addresses, transport to/from school could be identified and excluded. The output measures walking in leisure time (trips/day; min/day), cycling in leisure time (trips/day; min/day), passive transport in leisure time (trips/day; min/day) were computed in the PostgreSQL database. GPS-determined trips/day, minutes/day and minutes/trip were used as main outcomes and were stratified on week- and weekend-days for each transport mode.

### Data analysis

The Statistical Package for the Social Sciences for Windows version 21 (SPSS Inc., Chicago, IL, USA) was used to describe and analyze the characteristics of the sample. Means, standard deviations (SD) and percentages were used to describe the sample and to report GPS-determined and self-reported active (walking and cycling) and passive transport in leisure time.

Pearson correlations were calculated to examine the concurrent validity between GPS-determined and self-reported transport (walking, cycling, passive transport) in leisure time (trips/day), stratified on week- and weekend-days. Correlations were considered as low (≤0.30), moderate (0.31–0.50) and high (>0.50) [50]. T-tests were used to test differences between GPS-determined and self-reported transport in leisure time, stratified into week- and weekend-days, and to test differences of GPS-determined transport in leisure time between week- and weekend-days.

To determine the associations between the parental perceptions of the neighborhood environment and GPS-determined transport in leisure time, R version 3.03 was used. Three types of 2-level models were constructed (participants clustered within classes) using the LMER-function available in the lme4-package (http://cran.r-project.org/web/packages/lme4/index.html). Independent variables included all scales of the parental perceived neighborhood environmental attributes (residential density, land use mix access, street network connectivity, availability and quality of walking and cycling facilities, aesthetics, safety from traffic and safety from crime). The dependent variables were GPS-determined walking in leisure time (trips/day; min/day), cycling in leisure time (trips/day; min/day), passive transport in leisure time (trips/day; min/day), separated for week- and weekend-days. All dependent variables, except for GPS-determined walking during weekdays, were non-normally distributed. Since the dependent variable GPS-determined walking during weekdays (trips/day; min/day) was normally distributed, a first type of model (Gaussian model with link function ‘identity’) was used and fitted using maximum likelihood. Akaike’s Information Criterion (AIC) tests confirmed that a Gaussian model with link function ‘identity’ was the best model to fit these data. From this model, beta-coefficients and 95 % confidence intervals were reported. Since the other dependent variables for weekdays were non-normally distributed [GPS-determined cycling and passive transport during weekdays (trips/day; min/day)], Gamma models with link function ‘log’ were used. AIC tests confirmed that Gamma models with link function ‘log’ were the best models to fit these data. Exponents of b (proportional increase in the dependent variable with a one-unit increase in the independent variable) with 95 % confidence intervals were reported for the Gamma models.

The dependent variables during weekend days [GPS-determined walking, cycling and passive transport during weekend days (trips/day; min/day)] were non-normally distributed and had an excessive number of zeros. Therefore, generalized linear mixed hurdle models (GLMMs), adjusting for the clustering of participants within classes, were used with the GLMER-function in the lme4-package [51]. Within a hurdle model, two separate analyses are performed. First, logistic regression models (logit model) were run that estimate the associations between the independent variables and the odds of engaging in walking, cycling or passive transport during weekend days (1 or more trips). Second, Gamma models with link function ‘log’ were used to investigate the associations with parental perceptions of the neighborhood environment among those who walked, cycled or used passive transport during weekend days (=non-zeros). GLMMs were fitted by Adaptive Gauss-Hermite Quadrature with 25 quadrature points. Odds ratio (OR) with 95 % confidence intervals were reported for the logit models, exponents of b with 95 % confidence intervals were reported for the Gamma models. All analyses were controlled for age (continuous), sex, SES, wear time and school. The significance level was defined at 0.05.

## Results

### Description of study sample

Of the 126 children with valid accelerometer and GPS data, 64 % (n = 80) were girls. Fifty-two percent went to a suburban school (n = 65), the other 48 % (n = 60) to an urban school. In total, 75.2 % (n = 94) had a high SES. Mean age was 10.6 ± 0.6 years.

### GPS-determined versus self-reported transport in leisure time

In Table 1, GPS-determined and self-reported walking, cycling and passive transport in leisure time during week- and weekend-days are described. Trips/day, minutes/day, minutes/trip and percentages of children not engaging in walking, cycling and passive transport are shown in Table 1. Pearson correlations and differences between GPS-determined and self-reported walking, cycling and passive transport (trips/day) are reported in Table 1.

**Table 1:** GPS-determined and self-reported transport in leisure time during week- and weekend-days (n = 126)

	Weekday				Weekend day				GPS-determined difference week–weekend day
	GPS-determined	Self-reported	r	t-value	GPS-determined	Self-reported	r	t-value	t-value
*Walking*									
Trips/day (M ± SD)	3.96 ± 1.60	0.24 ± 0.45	0.03	25.39***	1.59 ± 1.60	0.47 ± 0.81	0.27**	8.03***	12.14***
Minutes/day (M ± SD)	26.10 ± 10.51				13.35 ± 17.20				7.32***
Minutes/trip (M ± SD)	6.83 ± 2.13				7.89 ± 4.84				−2.09*
No walking (n, (%))	0 (0.0)	80 (69.6)			31 (24.6)	73 (64.0)			
*Cycling*									
Trips/day (M ± SD)	1.17 ± 0.87	0.14 ± 0.46	0.25**	13.20***	0.87 ± 0.96	0.22 ± 0.51	0.30**	7.87***	2.94**
Minutes/day (M ± SD)	7.85 ± 7.46				5.91 ± 7.60				2.40*
Minutes/trip (M ± SD)	6.23 ± 2.78				6.67 ± 4.04				−0.87
No cycling (n, (%))	12 (9.5)	100 (87.0)			41 (32.5)	92 (80.7)			
*Passive transport*									
Trips/day (M ± SD)	1.87 ± 1.54	1.02 ± 0.82	0.57***	8.02***	2.12 ± 1.61	2.00 ± 1.39	0.59***	1.25	−1.50
Minutes/day (M ± SD)	16.37 ± 16.19				31.39 ± 33.77				−5.40***
Minutes/trip (M ± SD)	8.37 ± 3.64				15.90 ± 16.95				−4.17***
Not using passive transport (n, (%))	19 (15.1)	27 (23.5)			26 (20.6)	16 (14.0)			

*M* Mean, *SD* standard deviation, *r* Pearson correlation coefficient

* p < 0.05; ** p < 0.01; *** p < 0.001

The number of GPS-determined trips/day was significantly higher than the number of self-reported trips/day for walking during week—(t = 25.39; p < 0.001) and weekend-days (t = 8.03; p < 0.001), for cycling during week—(t = 13.20; p < 0.001) and weekend-days (t = 7.87; p < 0.001), and for passive transport during weekdays (t = 8.02; p < 0.001). No significant difference was found for passive transport during weekend days (t = 1.25, p = 0.22).

No significant correlation was found between GPS-determined and self-reported transport in leisure time for walking during weekdays. Low correlations between GPS-determined and self-reported measures were found for walking during weekend days (r = 0.27; p = 0.004), cycling during weekdays (r = 0.25; p = 0.007) and cycling during weekend days (r = 0.30; p = 00.002). High correlations between GPS-determined and self-reported measures were found for passive transport during weekdays (r = 0.57; p < 0.001) and during weekend days (r = 0.59; p < 0.001).

Compared to GPS-determined measures, higher self-reported percentages of not engaging in walking during week—(self-reported: 69.6 %—GPS: 0.0 %) and weekend-days (self-reported: 64.0 %—GPS: 24.6 %), cycling during week—(self-reported: 87.0 %—GPS: 9.5 %) and weekend-days (self-reported: 80.7 %—GPS: 32.5 %) and passive transport during weekdays (self-reported: 23.5 %—GPS: 15.1 %) were found. In contrast, a lower percentage was found for not engaging in self-reported passive transport during weekend days (14.0 %) compared to percentages determined by GPS (20.6 %).

### Differences of GPS-determined transport in leisure time between week- and weekend-days

Differences of GPS-determined transport in leisure time between week- and weekend-days are shown in Table 1. Children had significantly more trips/day and minutes/day of walking (trips/day: t = 12.14; p < 0.001, minutes/day: t = 7.32; p < 0.001) and cycling (trips/day: t = 2.94; p = 0.004, minutes/day: t = 2.40; p = 0.02) during weekdays compared to weekend days. In contrast, children engaged in significantly more minutes/trip of walking during weekend days (t = −2.09; p = 0.04). Significantly lower minutes/day (t = −5.40; p < 0.001) and minutes/trip (t = −4.17; p < 0.001) of passive transport were found during weekdays compared to weekend days. No significant difference was found for minutes/trip of cycling (t = −0.87, p = 0.39) and for trips/day of passive transport (t = −1.50, p = 0.14).

### Associations between parental perceptions of the neighborhood environment and GPS-determined transport in leisure time

The results of the final models for the associations between parental perceptions of the neighborhood environment and GPS-determined walking, cycling and passive transport during week- and weekend-days are shown in Table 2 (trips/day) and Table 3 (minutes/day).

**Table 2:** Associations between parental perceptions of the neighborhood environment and GPS-determined transport (in trips/day)

	Walking (trips/day) Week	Cycling (trips/day) Week	Passive transport (trips/day) Week
	Gaussian model (n = 126)	Gamma model (n = 126)	Gamma model (n = 126)
	β (95 % CI)	Exp b (95 % CI)^a^	Exp b (95 % CI)^a^
Residential density	0.01 (−0.01, 0.01)	1.00 (0.99, 1.00)	*0.99* (*0.99*, *1.00*)**
Land use mix access	−0.34 (−0.75, 0.08)	*1.13* (*1.01*, *1.27*)*	0.85 (0.73, 1.00)^(^*^)^
Street network connectivity	−0.03 (−0.53, 0.46)	1.14 (0.99, 1.31)^(^*^)^	1.10 (0.91, 1.32)
Walking and cycling facilities	0.11 (−0.34, 0.57)	*0.83* (*0.73*, *0.94*)**	0.94 (0.79, 1.12)
Aesthetics	0.36 (−0.17, 0.90)	0.96 (0.84, 1.11)	1.04 (0.85, 1.28)
Traffic safety	−0.24 (−0.64, 0.16)	0.99 (0.88, 1.10)	0.95 (0.81, 1.11)
Crime safety	0.25 (−0.07, 0.56)	1.13 (0.93, 1.34)	0.97 (0.87, 1.10)

	Walking (trips/day) Weekend		Cycling (trips/day) Weekend		Passive transport (trips/day) Weekend	
	Logistic model^b^ (n = 126)	Gamma model^c^ (n = 95)	Logistic model^b^ (n = 126)	Gamma model^c^ (n = 85)	Logistic model^b^ (n = 126)	Gamma model^c^ (n = 101)
	OR (95 % CI)	Exp b (95 % CI)^a^	OR (95 % CI)	Exp b (95 % CI)^a^	OR (95 % CI)	Exp b (95 % CI)^a^
Residential density	0.99 (0.96, 1.01)	1.00 (0.99, 1.02)	1.00 (0.97, 1.02)	1.00 (0.98, 1.01)	0.99 (0.96, 1.03)	1.00 (0.98, 1.01)
Land use mix access	1.30 (0.53, 3.21)	0.87 (0.53, 1.43)	0.94 (0.42, 2.10)	1.03 (0.64, 1.66)	0.48 (0.16, 1.47)	0.97 (0.65, 1.45)
Street network connectivity	1.03 (0.35, 2.98)	1.08 (0.60, 1.93)	0.81 (0.31, 2.11)	0.86 (0.47, 1.58)	1.22 (0.33, 4.50)	0.84 (0.51, 1.39)
Walking and cycling facilities	1.09 (0.39, 3.03)	*1.74 (1.07, 2.85*)*	1.25 (0.50, 3.16)	1.45 (0.81, 2.57)	1.16 (0.34, 3.92)	1.17 (0.76, 1.82)
Aesthetics	0.62 (0.19, 2.06)	1.33 (0.75, 2.34)	1.34 (0.44, 4.12)	0.74 (0.39, 1.40)	2.49 (0.61, 10.15)	0.84 (0.48, 1.45)
Traffic safety	1.53 (0.62, 3.77)	0.91 (0.56, 1.50)	0.83 (0.38, 1.82)	0.94 (0.57, 1.56)	0.88 (0.33, 2.35)	1.01 (0.68, 1.48)
Crime safety	1.14 (0.56, 2.31)	1.03 (0.73, 1.47)	0.65 (0.35, 1.22)	1.02 (0.76, 1.49)	1.70 (0.79, 3.68)	1.22 (0.90, 1.65)

*OR* odds ratio, *CI* confidence interval

italic = significant (p < 0.05)

** p < 0.01; * p < 0.05; ^(*)^ p < 0.10

All models were adjusted for age, sex, socio-economic status (SES), school and wear time

^a^Exp b = exponent of b, all Gamma models were fitted using a log link function, the exponent of the b’s can be interpreted as a proportional increase in the dependent variable (in trips/day) with a one-unit increase in the independent variable

^b^The logistic model estimates the associations between the independent variables and the odds of walking, cycling or using passive transport during weekend days

^c^The Gamma model estimates the associations between the independent variables and the amount of walking, cycling or passive transport during weekend days (in trips/day) among those who have walked, cycled and used passive transport during weekend days

**Table 3:** Associations between parental perceptions of the neighborhood environment and GPS-determined transport (in minutes/day)

	Walking (min/day) Week	Cycling (min/day) Week	Passive transport (min/day) Week
	Gaussian model (n = 126)	Gamma model (n = 126)	Gamma model (n = 126)
	β (95 % CI)	Exp b (95 % CI)^a^	Exp b (95 % CI)^a^
Residential density	*0.10* (*0.01, 0.19*)*	0.99 (0.99, 1.00)	0.99 (0.98, 1.00)^(^*^)^
Land use mix access	−2.04 (−5.12, 1.05)	*1.46* (*1.11, 1.92*)**	0.74 (0.53, 1.04)^(^*^)^
Street network connectivity	0.68 (−3.01, 4.37)	1.19 (0.86, 1.66)	1.12 (0.76, 1.64)
Walking and cycling facilities	0.50 (−2.88, 3.88)	*0.72* (*0.54, 0.97*)*	0.83 (0.57, 1.20)
Aesthetics	*4.69* (*0.75, 8.64*)*	1.03 (0.74, 1.43)	1.33 (0.86, 2.05)
Traffic safety	0.40 (−2.58, 3.39)	0.87 (0.68, 1.13)	0.99 (0.73, 1.35)
Crime safety	1.50 (−0.82, 3.81)	1.19 (0.89, 1.45)	1.00 (0.78, 1.28)

	Walking (min/day) Weekend		Cycling (min/day) Weekend		Passive transport (min/day) Weekend	
	Logistic model^b^ (n = 126)	Gamma model^c^ (n = 95)	Logistic model^b^ (n = 126)	Gamma model^c^ (n = 85)	Logistic model^b^ (n = 126)	Gamma model^c^ (n = 101)
	OR (95 % CI)	Exp b (95 % CI)^a^	OR (95 % CI)	Exp b (95 % CI)^a^	OR (95 % CI)	Exp b (95 % CI)^a^
Residential density	0.99 (0.96, 1.01)	1.01 (0.99, 1.02)	1.00 (0.97, 1.02)	1.00 (0.98, 1.01)	0.99 (0.96, 1.03)	1.00 (0.98, 1.01)
Land use mix access	1.30 (0.53, 3.21)	0.93 (0.56, 1.54)	0.94 (0.42, 2.10)	1.31 (0.77, 2.20)	0.48 (0.16, 1.47)	0.96 (0.64, 1.44)
Street network connectivity	1.03 (0.35, 2.98)	1.06 (0.60, 1.89)	0.81 (0.31, 2.11)	0.64 (0.35, 1.18)	1.22 (0.33, 4.50)	0.85 (0.52, 1.40)
Walking and cycling facilities	1.09 (0.39, 3.03)	*1.92* (*1.14*, *3.26*)*	1.25 (0.50, 3.16)	1.60 (0.86, 2.95)	1.16 (0.34, 3.92)	1.15 (0.74, 1.79)
Aesthetics	0.62 (0.19, 2.06)	1.25 (0.69, 2.25)	1.34 (0.44, 4.12)	0.71 (0.37, 1.39)	2.49 (0.61, 10.15)	0.84 (0.48, 1.45)
Traffic safety	1.53 (0.62, 3.77)	0.87 (0.52, 1.45)	0.83 (0.38, 1.82)	0.80 (0.46, 1.39)	1.14 (0.43, 3.07)	1.02 (0.69, 1.51)
Crime safety	1.14 (0.56, 2.31)	1.21 (0.84, 1.73)	0.65 (0.35, 1.22)	0.94 (0.65, 1.38)	0.59 (0.27, 1.27)	1.21 (0.89, 1.64)

All models were adjusted for age, sex, socio-economic status (SES), school and wear time

*OR* odds ratio, *CI* confidence interval

Italic = significant (p < 0.05)

** p < 0.01; * p < 0.05; ^(*)^ p < 0.10

^a^Exp b = exponent of b, all Gamma models were fitted using a log link function, the exponent of the b’s can be interpreted as a proportional increase in the dependent variable (in minutes/day) with a one-unit increase in the independent variable

^b^The logistic model estimates the associations between the independent variables and the odds of walking, cycling or using passive transport during weekend days

^c^The Gamma model estimates the associations between the independent variables and the amount of walking, cycling or passive transport during weekend days (in minutes/day) among those who have walked, cycled and used passive transport during weekend days

### Trips per day

No significant associations were found for walking trips per day during weekdays. The Gamma model showed that more cycling trips/day during weekdays were performed when a higher land use mix access was perceived by the parents (Exp b = 1.13). Additionally, less cycling trips/day during weekdays were performed when more and better walking or cycling facilities were perceived by the parents (Exp b = 0.83). The Gamma model showed that less passive trips/day during weekdays were performed when a higher residential density was perceived by the parents (Exp b = 0.99).

None of the logistic models showed significant associations with parental perceptions of the neighborhood environment and the odds of walking, cycling or use of passive transport during weekend days.

The Gamma model showed that among those who walked during weekend days, more walking trips/day were performed when more and better walking or cycling facilities were perceived by the parents (Exp b = 1.74). Furthermore, no further associations were found.

### Minutes per day

The Gaussian model showed significant positive associations with residential density and with aesthetics for minutes walking per day during weekdays. Children walked more minutes per day during weekdays when a higher residential density (β = 0.10) and better aesthetics (β = 4.69) of the neighborhood were perceived by the parents. The Gamma model showed that more min/day of cycling during weekdays were performed when a higher land use mix access was perceived by the parents (Exp b = 1.46). Additionally, less min/day of cycling during weekdays were performed when more and better walking or cycling facilities were perceived by the parents (Exp b = 0.72).

None of the logistic models showed significant associations with parental perceptions of the neighborhood environment and the odds of walking, cycling or use of passive transport during weekend days.

The Gamma model showed that among those who walked during weekend days, more min/day of walking were performed when more and better walking or cycling facilities were perceived by the parents (Exp b = 1.92). Furthermore, no further associations were found within the Gamma model.

## Discussion

Overall, the results showed that children under-reported their walking and cycling in leisure time during week- and weekend-days compared to GPS-determined walking and cycling, which confirms our hypothesis. Under-reporting was found for both trips/day of walking or cycling and percentages of not engaging in walking or cycling. A remarkable finding was that about 70 % of the children reported to not engage in walking during weekdays, while GPS-determined measures of walking showed that all children walked to leisure-time destinations. Similar to the results of walking, children’s GPS-determined cycling was a lot higher compared to self-reported measures of cycling. Studies comparing children’s GPS-determined and self-reported transport are scarce [36, 37]. Consistent with our results, Mackett et al. [36] found under-reporting of children’s self-reported trips. However, in the literature no distinction was made between different (active and passive) transport modes and trips were not specifically defined for children’s leisure time. Under-reporting of self-reported walking and cycling trips may be due to the fact that children (and parents) may forget to report short and occasional trips of walking and cycling. Rodriguez et al. [38] demonstrated in adolescents that it was difficult to report short active trips being part of a trip chain (e.g. walking trip to bus stop not reported), and that reporting their transport over multiple days could led to negligence resulting in less self-reported active transport [38, 39]. While children under-reported their active trips, the results of the current study indicated that children reported their passive transport during weekend days quite accurate. The moderate correlations between GPS-determined and self-reported passive transport for week- and weekend-days also demonstrated that children had less difficulties to report their passive transport behavior in leisure time. Rodriguez et al. [38] stated that car trips are usually longer and therefore easier to remember than active trips. Based on the findings of the present study, it may be recommended for research examining children’s active transport in leisure time to use GPS. Using GPS provides many advantages to assess children’s active transport in leisure time: it is an objective method, valid and user friendly instrument to use among children [46], a distinction between different transport modes can be made and the exact context-specific behavior can be obtained. Researchers should however be aware that signal loss, short battery life and children forgetting to recharge the GPS sometimes leads to less accurate measures [36, 37, 42].

When examining children’s GPS-determined transport in leisure time, walking was the most frequently performed transport mode during weekdays and passive transport during weekend days. Previous studies using GPS to report measures of children’s transport in leisure time are scarce [33, 35, 39] and only one of those studies reported separate results for week- and weekend-days [35]. Notwithstanding different reporting of results in previous studies compared to our study (e.g. no distinction between walking and cycling, not specifically reporting on children’s transport in leisure time), our findings of walking or cycling and passive transport are higher compared to the active transport (ranging from 18.8 to 30.5 min/day) and passive transport rates (ranging from 2.1 to 11.3 min/day) found in previous studies. Furthermore, our finding that children walked remarkably more during weekdays compared to weekend days could be explained by the fact that children spend more time inside during the weekend and travel less frequently to leisure-time destinations [52]. An explanation for the finding that children used more passive transport on weekend days could be that children travel to other leisure-time activities during weekends [7, 18] and that larger distances have to be traveled, resulting in more frequently using passive transport during weekend days [53]. Those findings confirm our hypothesis that GPS-determined transport in leisure time differs between week- and weekend-days. Additionally, GPS-determined number of trips/day and minutes/day of cycling among children in the present study were rather low and small differences of cycling between week- and weekend-days were found. A reason for the fact that we found that children engaged more frequently in walking compared to cycling could be that short trips were included in our GPS-determined measures and that many walking trips tend to be short, as previously described by Rodriguez and colleagues (2012) [38]. It could be that short trips are relevant to children’s overall health, so it is of interest that future studies investigate if these short active trips have an influence on children’s health outcomes. Based on our GPS-determined findings, it can be recommended to promote active transport in weekend days, but also other types of physical activity, since it is known that total physical activity is overall lower on weekend days [54, 55].

Concerning the second aim of the present study, the results indicated that only few parental perceived environmental attributes of the neighborhood were associated with children’s GPS-determined walking, cycling and passive transport in leisure time. Consistent with findings of previous studies, although using self-reported measures of active transport in leisure time, [8, 9, 18, 19, 21, 23], we found a positive association between residential density and minutes walking during weekdays (and a negative association for passive transport during weekdays), a positive association between land use mix access and cycling during weekdays, and no associations for safety from traffic and crime. Furthermore, studies in the past reported inconclusive results regarding the contribution of parental perceived neighborhood aesthetics and walking and cycling facilities [9]. In our study, we found a positive association between perceived aesthetics and children’s minutes of walking per day during weekdays. The presence of green space was previously identified as an important determinant for children’s active transport [16, 17], which partially could explain our finding. Surprisingly, contrasting results were found for quality and availability of walking and cycling facilities. More and better walking and cycling facilities were associated with more walking during weekend days, but were also associated with less cycling during weekdays. No plausible explanation was found for these contrasting findings concerning the association between walking and cycling facilities and active transportation. It is possible that other factors than walking and cycling facilities are more important to explain children’s active transport (e.g. residential density, land use mix access, family and friend support).

The present study has important strengths. Until now, other studies assessing children’s transport specifically during leisure time relied on subjective recall. To our knowledge, this is the first study using GPS to determine transport in leisure time during both week- and weekend-days and adding children’s self-reported measures to compare with children’s GPS-determined transport in leisure time. Additionally, this is the first study using this objective method to examine the associations between children’s GPS-determined transport in leisure time and parental perceptions of the neighborhood environment. However, future research using GPS with a focus on children’s transport in leisure time is needed to confirm and elaborate the results of the present study and this across other populations (e.g. younger children). Other strengths of this study were the selection of both suburban and urban schools across Flanders and the measurement period over multiple days (7-days including week- and weekend-days) which induces high reliability [56].

Some limitations of this study should be considered. The cross-sectional character of the study is a limitation, as no causal relationships between the parental perceptions of the neighborhood and children’s transport in leisure time can be examined. Another limitation involved the relatively small sample size, which limits power and generalizability. Third, the used algorithms to detect trips and classify trip modes are relatively simplistic and are found to misclassify 20–25 % of the trips and trip modes [49]. Future studies could benefit from improved trip detection and trip mode classification. Children of low SES were underrepresented and the findings are also only generalizable for (sub-)urban areas of Flanders. Fourth, data collection was conducted during winter, and therefore it is unknown if the results are generalizable to the other seasons [35, 57]. However, Flanders is characterized by mild winters. At last, only parental perceptions of the neighborhood environment were examined with children’s transport in leisure time. Since the interactions between individual, social and environmental factors make it difficult to examine the exact relation between the neighborhood environment and children’s transport in leisure time, future research should include the effect of individual and social factors.

## Conclusions

First, the current study demonstrated that 10 to 12-year old children tend to under-report their walking and cycling in leisure time and yet report their passive transport during weekend days quite accurate. Based on GPS-determined data, we observed that children walked most during weekdays and used more frequently passive transport during weekend days. Only few parental perceived environmental attributes of the neighborhood (i.e. residential density, land use mix access, quality and availability of walking and cycling facilities, and aesthetics) were significantly associated with children’s GPS-determined walking, cycling or passive transport in leisure time. In conclusion, to accurately assess children’s active transport in leisure time, GPS use is recommended. Additionally, more research using GPS with a focus on children’s transport in leisure time and investigating the associations with parental perceptions of the neighborhood environment is needed to confirm and elaborate the results of the present study.

## Additional file

10.1186/s12942-016-0045-9 Outline of questionnaire to assess parental perceptions of the neighborhood environment. This file provides an outline of the questionnaire to assess parental perceptions of the neighborhood environment, respectively the questions for the subscales with corresponding response options. ^1^scored on a 5-point scale (none, a few, some, most, all); ^2^scored on a 4-point scale ranging from strongly disagree to strongly agree; ^a^questions deriving from parent version of NEWS-Y (Rosenberg et al. 2009 [9]).
