# Supplementary material for: Children’s GPS-determined versus self-reported transport in leisure time and associations with parental perceptions of the neighborhood environment
Source: Int J Health Geogr. 2016 May 5;15:16. doi: 10.1186/s12942-016-0045-9 (PMC4858916; doi:10.1186/s12942-016-0045-9)
Supplement: Supplementary file 1 — 10.1186/s12942-016-0045-9 Outline of questionnaire to assess parental perceptions of the neighborhood environment. This file provides an outline of the questionnaire to assess parental perceptions of the neighborhood environment, respectively the questions for the subscales with corresponding response options. 1scored on a 5-point scale (none, a few, some, most, all); 2scored on a 4-point scale ranging from strongly disagree to strongly agree; aquestions deriving from parent version of NEWS-Y (Rosenberg et al. 2009 [9]). [file 12942_2016_45_MOESM1_ESM.docx]

| **Additional file 1:**  **Outline of questionnaire to assess parental perceptions of the neighborhood environment** | |
| --- | --- |
|  | |
| ***Residential density^1^*** |  |
| 1a. How common are separate or standalone one family homes in your neighborhood?^a^ | |
| 1b. How common are connected townhouses or row houses in your neighborhood?^a^ | |
| 1c. How common are apartments in your neighborhood?^a^ | |
| ***Land use mix access^2^*** | |
| 2a. There are many places for my child to go (alone or with someone) within easy walking distance of our home.^a^ | |
| 2b. From our home, it is easy for my child to walk to a transit stop (bus, subway, train).^a^ | |
| 2c. There are major barriers to walking in our local area that make it hard for my child to get from place to place (for example, freeways, railway lines, rivers).^a^ | |
| 2d. In my neighbourhood it’s easy for my child to walk to a playground, park or skate park from my house. | |
| ***Street network connectivity^2^*** | |
| 3a. The streets in our neighborhood have many cul-de-sacs (dead end streets).^a^ | |
| 3b. There are a lot of crossroads in my neighborhood.^a^ | |
| 3c. There are many different routes for getting from place to place in our neighborhood (my child doesn’t have to go the same way every time).^a^ | |
| ***Walking and cycling facilities^2^*** | |
| 4a. There are sidewalks on most of the streets in our neighborhood.^a^ | |
| 4b. There are cycle lanes on most of the streets in our neighborhood. | |
| 4c. Sidewalks are separated from the road/traffic in our neighborhood by parked cars or grass.^a^ | |
| 4d. Cycle lanes are separated from the road/traffic in our neighborhood by parked cars or grass. | |
| 4e. At night the sidewalks are well-lit in my neighborhood. | |
| 4f. At night the cycle lanes are well-lit in my neighborhood. | |
| 4g. The sidewalks are well maintained in my neighborhood. | |
| 4h. The cycle lanes are well maintained in my neighborhood. | |
| ***Aesthetics^2^*** | |
| 5a. There are trees along the streets in our neighborhood.^a^ | |
| 5b. There is not much litter or graffiti in my neighborhood. | |
| 5c. There are many beautiful natural things for my child to look at in my neighborhood (e.g. gardens, views).^a^ | |
| 5d. There are many buildings/homes in our neighborhood that are nice for my child to look at.^a^ | |
| 5e. The playgrounds, parks and other open spaces where children can play are well maintained in my neighborhood. | |
| ***Traffic safety^2^*** | |
| 6a. There is so much traffic along nearby streets that it makes it difficult or unpleasant for my child to walk (alone or with someone) in our neighborhood.^a^ | |
| 6b. There is so much traffic along nearby streets that it makes it difficult or unpleasant for my child to cycle (alone or with someone) in our neighborhood. | |
| 6c. The speed of traffic on most nearby streets is usually slow (50 km/h or less).^a^ | |
| 6d. There are crosswalks and signals to help walkers cross busy streets in our neighborhood.^a^ | |
| 6e. It’s safe for my child to play on the street in my neighborhood. | |
| ***Crime safety^2^*** | |
| 7a. I am worried about letting my child play outside alone around my home (e.g. yard, driveway, apartment common area) because I am afraid of then being taken or hurt by a stranger.^a^ | |
| 7b. I am worried about letting my child be alone or with friends in a local or nearby park because I am afraid my child will be taken or hurt by a stranger.^a^ | |
|  | |
